# Jumping on the ‘bad’wagon? How group membership influences responses to the social exclusion of others

**DOI:** 10.1093/scan/nsaa070

**Published:** 2020-05-21

**Authors:** Gert-Jan Lelieveld, Lasana T Harris, Lotte F van Dillen

**Affiliations:** 1 Department of Social, Economic, and Organizational Psychology, Institute of Psychology, Leiden University, Leiden 2333AK, the Netherlands; 2 Leiden Institute for Brain and Cognition, Leiden 9600, the Netherlands; 3 Department of Experimental Psychology, University College London, London WC1H 0AP, UK

**Keywords:** social exclusion, fMRI, group membership, dlPFC, Cyberball

## Abstract

In four studies, we addressed whether group membership influences behavioral and neural responses to the social exclusion of others. Participants played a modified three-player Cyberball game (Studies 1–3) or a team-selection task (Study 4) in the absence or presence of a minimal group setting. In the absence of a minimal group, when one player excluded another player, participants actively included the excluded target. When the excluder was from the in-group and the excluded player from the out-group, participants were less likely to intervene (Studies 1–3) and also more often went along with the exclusion (Study 4). Functional magnetic resonance imaging results (Study 3) showed that greater exclusion in the minimal group setting concurred with increased activation in the dorsolateral pre-frontal cortex, a region associated with overriding cognitive conflict. Self-reports from Study 4 supported these results by showing that participants’ responses to the target’s exclusion were motivated by group membership as well as participants’ general aversion to exclude others. Together, the findings suggest that when people witness social exclusion, group membership triggers a motivational conflict between favoring the in-group and including the out-group target. This underscores the importance of group composition for understanding the dynamics of social exclusion.

## Jumping on the ‘bad’wagon? How group membership influences responses to the social exclusion of others

Previous research on social exclusion strongly focused on its detrimental effects for victims (Williams, 2007), but the answer to the question why people exclude others and under which circumstances remains inconclusive (e.g. Wesselmann *et al*., 2013). The scarce research on the decision to include *vs* exclude has shown that inclusion is the norm in most social situations (Kerr *et al*., 2008) and that explicit instructions to ostracize others induce emotional distress (Zadro *et al*., 2005). Still, social exclusion occurs frequently among both children (Wang *et al*., 2010) and adults (Williams, 2007), underscoring the need to better understand the factors driving social exclusion. In this light, it is important to consider that exclusion is typically a group effort. To understand the dynamics of social exclusion, it is thus important to incorporate this group context and not only focus on the initiator of exclusion but also examine how others within the group react in turn. These other group members might intervene by trying to again include the excluded target, observe the situation without addressing the exclusion or actively go along with the exclusion (Malti *et al*., 2015). Different motives may underlie the decision to intervene or not, such as the motivation to include the excluded target because of exclusion aversion and/or the motivation to favor the person who initiated the exclusion. These motives are not mutually exclusive and can create a dilemma for people when deciding how to respond to the exclusion of a target. The main goal of the current research was to study whether social inclusion norms toward the target could emerge without group conformity norms to reciprocate the excluder. Thus, group membership may play a central role in encouraging the decision to include the target or favor the excluder.

There are many arguments for why group membership should affect social exclusion. Prior research has shown that in-group members are seen as more similar in attitudes and values than out-group members and that this shapes our social interactions (Tajfel and Turner, 1986). People have more affinity for in-group members and tend to favor them over out-group members (Hewstone *et al*., 2002). Out-group members, compared to in-group members, elicit less trust (Voci, 2006). Moreover, we grant fewer resources (Tajfel *et al*., 1971) and offer less help to out-group members (Levine *et al*., 2002). Group membership may thus be a key determinant of social exclusion dynamics.

Surprisingly, research has so far mainly considered the effects of group membership on ‘victims’ of social exclusion. This research has shown that social exclusion leads to pain and distress, regardless of whether it was initiated by an in-group or out-group member (Williams *et al*., 2000; Smith and Williams, 2004) or even a strongly disliked out-group member (Gonsalkorale and Williams, 2007, although see Wirth and Williams, 2009; Bernstein *et al*., 2010; Goodwin *et al*., 2010, for a more nuanced perspective). Little research, however, has addressed the effects of group membership on the process of exclusion itself. In one exception, Vrijhof *et al.* (2016) examined responses to the social exclusion of in-group and out-group members among adolescents. They found that adolescents generally applied a strong inclusion norm; they actively tried to include both in-group and out-group members, even though adolescents’ empathic concern was associated more with the inclusion of in-group members than with the inclusion of out-group members. This study provided a first step in examining the effects of group membership on the process of exclusion, but with mixed results for adolescents’ motives *vs* actual behavior. It is moreover still an open question how group membership affects reactions to social exclusion among adults or what the underlying (brain) mechanisms are for different reactions to social exclusion. To address this hiatus, in the first three studies, we investigated people’s behavioral and brain responses to the exclusion of another individual using a modified three-player Cyberball game (a computerized ball-tossing game; Williams *et al*., 2000). Exclusion was programmed such that one player (the excluder) threw the ball consistently to the participant at the cost of another player (the excluded target). To manipulate group membership, we used a minimal group paradigm, which creates groups based on arbitrary dimensions, thereby reducing any bias from existing knowledge about specific social groups (Tajfel, 1970). In this minimal group, the excluder was always the in-group and the excluded target the out-group. By manipulating group membership through self-selection (Study 1) as well as random assignment (Studies 2 and 3), we moreover tested the robustness of its effects.

We chose the perspective of a group member that does not initiate the exclusion but responds to the exclusion initiated by another group member, because bullying research has shown that such facilitatory actions substantially reinforce the negative experience of bullying (Espelage *et al*., 2007). Also, people who observe bullying of out-group victims, compared to in-group victims, hold less negative attitudes toward in-group aggressors (Nesdale *et al*., 2013) and less often intervene (Palmer *et al*., 2015). In the current research, we addressed whether similar dynamics apply to social exclusion. We reasoned that group members could react to social exclusion in three ways: (1) by going along with the exclusion of the target (by reducing the number of throws to the excluded player), (2) by compensating and actively including the target (increasing throws to the excluded player) or (3) by doing neither (dividing tosses equally among the two players). Whereas the latter (on-the-fence) option does not involve an active exclusion, it could still be considered facilitatory since the excluded player ends up receiving fewer balls than equal distribution norms propose.

We moreover predicted participants’ responses to the social exclusion of another individual to be determined by the salience of the players’ group membership, something we addressed by means of our minimal group manipulation. In the absence of such information, we expected participants to compensate because of the strong inclusion norm (Zadro and Gonsalkorale, 2014). However, when we make group membership salient, and when an in-group member initiates the exclusion of an out-group member, we expected that this could create a dilemma in participants between favoring the in-group and avoiding the exclusion of the out-group target. That is, whereas participants may feel it is normative to not exclude others (Wesselmann *et al*., 2013), people may at the same time wish to reciprocate and favor the exclusionary behavior of the in-group member (Gaertner and Insko, 2000). As a consequence, we expected participants to remain unbiased and divide tosses equally among the two players.

This motivational conflict is likely moderated by one’s identification with one’s in-group (Akerlof and Kranton, 2000), such that the stronger this identification, the more participants are inclined to reciprocate an in-group excluder as opposed to compensating an excluded out-group target. To examine this, we additionally assessed participants’ felt connection with the in-group *vs* out-group player in the minimal group setting and examined their relation with participants’ tossing behavior, with greater relative in-group identification predicted to concur with reduced compensation.

In addition, in Study 3, we assessed to what extent cognitive conflict concurred with the activation of the two opposing motives (i.e. to include the out-group *vs* to favor the in-group) when participants witnessed social exclusion in a minimal group setting. Because this conflict is not necessarily expressed in people’s ultimate choices for exclusion *vs* inclusion, and because people are not always able to report on the conflict they experience while making a decision, we used functional magnetic resonance imaging (fMRI) to assess participants’ real-time brain indices of cognitive conflict during the Cyberball game. A large body of neuroimaging literature points to the central role of the dorsal anterior cingulate cortex (dACC) and the dorsolateral pre-frontal cortex (dlPFC; Van Veen and Carter, 2006; Botvinick, 2007) across tasks involving simple stimulus-response rules (Van Veen and Carter, 2005), as well as more complex social dynamics, like moral dilemmas (Greene *et al*., 2004), unethical behavior (Lelieveld *et al*., 2016) and social rejection (Somerville *et al*., 2006). Accordingly, these were our regions of interest (ROI) to test the assertion that a minimal group setting induces greater conflict resulting from the inclusion norm toward the out-group member competing with the norm to favor the in-group member.

In the first three studies, we used the Cyberball paradigm to examine people’s responses to the social exclusion of others. In a final study (Study 4), we investigated people’s responses to social exclusion with another paradigm, to see whether the findings of the first three studies translate to a different group setting that involved a team-selection task. In this paradigm (adjusted from Doolaard *et al*., 2020), participants are instructed to perform an estimation task in a team of four members. Following a practice round but before the actual game begins, participants are given the opportunity to adjust the composition of their team by excluding one of the other three players. This paradigm enabled us to study social exclusion in a larger group (i.e. a group of four). Moreover, in addition to the gradual act of social exclusion in the first three studies, operationalized as the relative number of throws to each other player, this paradigm allowed us to study how group membership affects the binary decision to include or exclude a member of the team. Finally, in Study 4 we assessed different motives underlying participants’ responses to social exclusion using self-report measures, to further examine whether people experienced conflict between the norm for inclusion and in-group-favoritism.

In four studies, involving behavioral as well as brain measures, we thus investigated the effect of group membership on compensating *vs* facilitating social exclusion of others and the dilemma people may experience when deciding between these two options. The first two studies involved a between-participant manipulation of inclusionary status (inclusion *vs* exclusion) and group membership (minimal group *vs* control) within an adjusted version of the Cyberball game. We thus examined how people respond to the exclusion of an individual in the absence *vs* presence of a minimal group setting. In the third neuroimaging study, we extended this setup and used an fMRI-compatible experimental design with inclusionary status as within-subjects factor and group membership as between-subjects factor. This allowed us to study the brain mechanisms underlying people’s reactions to social exclusion in the presence and absence of a minimal group. In a final study, we examined participants’ responses to social exclusion in a different group setting where people could adjust the composition of a team, using a between-participant manipulation of the order of the exclusion decision (initiating *vs* responding to the decision) and group membership (minimal group *vs* control).

## Study 1

### Method

#### Design and participants

The study used a 2 (inclusionary status, inclusion *vs* exclusion) × 2 (group membership, minimal group *vs* control) between-participants design. Our sample size was determined based on a power analysis revealing that 128 participants were required to detect a medium effect size (Cohen’s *d* = 0.50) at the 5% level with a power of 0.80. One hundred twenty-six undergraduates from Leiden University (75 women, 51 men; *M*_age_ = 20.88, SD_age_ = 2.31) eventually participated. They were recruited from the faculties of humanities, medicine, law, and science, but not from the faculty of social sciences, to ensure unfamiliarity with Cyberball. Participants were randomly assigned to the four conditions. All materials and datasets used in our studies are publicly available on the Open Science Framework, using the following link: https://osf.io/39gxr/?view_only=57d747f50cad4dc7bc0c1caf770d1f67.

### Procedure

Upon arrival at the laboratory, participants were led to a cubicle and received further instructions via the computer screen. Participants played a three-player Cyberball game (Williams *et al*., 2000), a computerized ball-tossing game. Originally, the game was programmed such that the participant was the exclusion target and would at some point stop receiving the ball. We modified this setup, such that now one of the virtual players was excluded by having the other player throw *all* balls to the participant (for a similar version, see Riem *et al*., 2013).

Participants were told they were to play a game of Cyberball with two others over the Internet. They learned that they were Player C, and the others were Players A and B. Participants were unaware that the behavior of the other players was pre-programmed. In the inclusion condition, Player A was programmed to throw the ball 50% of the times to the participant (Player C) and 50% of the times to Player B. In the exclusion condition, Player A threw the ball 100% of the times to the participant, thereby fully excluding Player B. In all conditions, Player B was programmed to at all times throw the ball 50% of the time to the participant and 50% of the time to Player A, thus displaying no biased behavior toward any of the other two players. The percentage of ball tosses from the participant to Player B (the exclusion target) comprised our main dependent variable. We calculated this as 100 (*N*_CB_/*N*_Total_), where *N*_CB_ is the number of throws from the participant to Player B and *N*_Total_ is the total number of throws. Both games proceeded for 45 throws in total (from all three players).

**Fig. 1:**
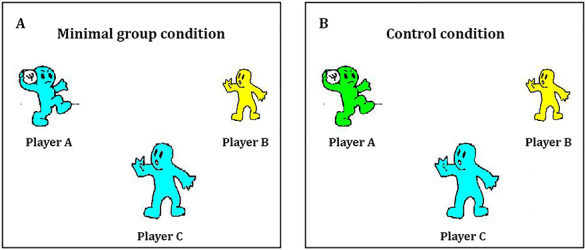
The group membership manipulation used in Studies 1–3. Participants in the minimal group condition (Figure 1A) played a game of Cyberball where they (Player C) had the same color as the person initiating the exclusion (Player A), but the target (Player B) had a different color. Participants in the control condition (Figure 1B) played a game where all three players had different colors.

Before the game started, participants chose their avatar to be blue, yellow, or green as a basis for our minimal group manipulation. In the minimal group condition, Player A’s color was matched to the participant’s choice, whereas Player B was given a different color. For instance, if the participant chose the color blue, Player A would also be blue, but player B would be yellow (see Figure 1A). In the control condition where group membership was absent, all players were assigned a different color. For instance, if a participant chose the color blue, Player A would be green and Player B yellow (see Figure 1B).

Following Cyberball, participants received a manipulation check of our minimal group manipulation. Participants indicated for Players A and B separately how connected they felt to them (i.e. ‘To what extent did you feel connected to Player A/B?’, ‘I had a lot in common with Player A/B’, and ‘Player A/B was a member of my group’), all on seven-point Likert scales (1 = ‘not at all’; 7 = ‘very strongly’). We averaged responses into a single index of perceived group membership (*α*_Player A_ = 0.80; *α*_Player B_ = 0.78). See the supplemental material for other questions we asked, including results.

Participants next estimated the number of tosses Player B had received in both the inclusion and exclusion games, as a manipulation check of our exclusion manipulation. At the end of the session, participants were debriefed, were paid €1.50 and thanked for their participation. All procedures were approved by the ethical committee of the Leiden University Institute of Psychology.

## Results

### Manipulation checks

#### Perceived group membership

A 2 (inclusionary status) × 2 (group membership) analysis of variance (ANOVA) on the perceived group membership ratings of Player A yielded only a main effect of group membership, *F*(1, 122) = 9.01, *P* = 0.003, partial η^2^ = 0.07, indicating that independent of whether participants took part in an inclusion game or an exclusion game, participants identified more with Player A when they both had the same color (*M* = 4.36, SD = 1.50), rather than when they had a different color (*M* = 3.54, SD = 1.53). The 2 × 2 ANOVA of the perceived group membership ratings of Player B also only revealed a main effect of group membership, *F*(1, 122) = 6.05, *P* = 0.015, partial η^2^ = 0.05, indicating that when only Player B had a different color, participants identified less with Player B (*M* = 3.25, SD = 1.28) than when all players had a different color (*M* = 3.86, SD = 1.47). Together, these results confirm that our minimal group manipulation was effective.

#### Perceived exclusion of target

A 2 × 2 ANOVA only showed a main effect of inclusionary status, *F*(1, 122) = 46.60, *P* < 0.001, partial η^2^ = 0.28. Participants thought Player B received fewer balls in the exclusion game (*M* = 12.19, SD = 3.07) than in the inclusion game (*M* = 15.52, SD = 2.42), confirming the effectiveness of the exclusion manipulation.

#### Ball tosses to target

A 2 × 2 ANOVA showed a significant main effect of inclusionary status, *F*(1, 122) = 12.53, *P* = 0.001, partial η^2^ = 0.09, qualified by a significant interaction, *F*(1, 122) = 6.35, *P* = 0.013, partial η^2^ = 0.05 (see Table 1 for means and standard deviations (s.d.)). In the inclusion games, the number of tosses in the group membership conditions did not differ, *t*(122) = −.68, *P* = 0.500, Cohen’s *d* = 0.20, 95% CI [−0.07–0.03], suggesting that group membership alone did not affect the number of throws to the two other players. But in the exclusion games, group membership did influence participants’ ball-tossing behavior; they less frequently tossed the ball to the excluded target in the minimal group condition than in the control condition, *t*(122) = 2.90, *P* = 0.004, Cohen’s *d* = 0.64, 95% CI [0.02–0.12], as predicted. Moreover, participants actively compensated for the target’s exclusion in the control condition, with their percentage of tosses toward the target significantly exceeding the even distribution of 50%, *t*(33) = 6.51, *P* < 0.001, Cohen’s *d* = 1.22, 95% CI [0.07–0.14]. Such compensation was not observed in the minimal group condition, *t*(29) = 1.42, *P* = 0.165, Cohen’s *d* = 0.23, 95% CI [−0.01–0.08].

**Table 1:** Percentage of ball tosses to the excluded player as a function of inclusionary status and group membership (Study 1)

	Inclusion		Exclusion	
	*M*	SD	*M*	SD
Minimal group	51.54%^a^	9.36	53.35%^a^	12.90
Control	49.84%^a^	7.35	60.57%^b^	9.46

*Note*: Means with different superscripts differ significantly across all cells (*P*s < 0.05, analyzed with simple-effect analyses).

#### Group identification and tosses to the target

To examine whether the strength of identification with in-group Player A relative to out-group Player B was associated with participants’ tossing behavior in the minimal group condition, we correlated difference scores between the perceived group membership of Player A and Player B with the percentage of ball tosses to Player B. For our hypotheses, it was important to use the difference score, instead of the identification with Players A and B separately, as it allowed us to investigate the throwing behavior of participants who were the most affected by our manipulation (and thus identified more with Player A while at the same time less with Player B). This correlation was significantly negative in the minimal group condition (*r* = −0.18, *P* = 0.049), indicating that the stronger the felt connection with the in-group excluder relative to the out-group target, the fewer balls participants threw to the excluded target. This correlation was non-significant in the control condition (*r* = −.09, *P* = 0.463).

## Discussion

Study 1 provided initial evidence that a simple minimal group manipulation made participants compensate less for the exclusion of a target in Cyberball, whereas they actively compensated for the exclusion in the absence of a minimal group setting. The more participants identified with the excluder than the excluded target in a minimal group setting, the less likely they were to compensate.

To replicate the results of Study 1, and to rule out that shared preferences drove group membership perceptions (Festinger, 1957) due to the overlap of their color preferences with those of another player, in a second study, the Cyberball players were assigned a color, instead of choosing this themselves (see Dunham *et al*., 2011), providing an even more stringent test of the notion that a minimal group setting affects reactions to the social exclusion of others.

## Study 2

### Method

#### Design and participants

The study again involved a 2 (inclusionary status, inclusion *vs* exclusion) × 2 (group membership, minimal group *vs* control) between-participants design. Using similar selection criteria as in Study 1, we aimed for the inclusion of 128 participants. One hundred twenty-two undergraduates from Leiden University (84 women, 38 men; *M*_age_ = 20.63, SD_age_ = 2.19) comprised the final sample and were randomly assigned to the four conditions.

#### Procedure

The procedure was similar to Study 1, except that the players’ colors were now pre-programmed by the experimenter, without any accompanying information. As depicted in Figure 1, the participant (Player C) was always assigned the color blue and Player B the color yellow. In the minimal group condition, Player A was assigned the same color as the participant (i.e. blue) and a different color than both other players (i.e. green) in the control condition.

After playing the Cyberball game, participants responded to the same three items from Study 1 measuring perceived group membership of Player A (*α* = 0.83) and Player B (*α* = 0.76) and their estimated number of throws to Players A and B.

## Results

### Manipulation checks

#### Perceived group membership

A 2 (inclusionary status) × 2 (group membership) ANOVA of perceived group membership ratings of Player A yielded only a main effect of group membership, *F*(1, 118) = 6.31, *P* = 0.013, partial η^2^ = 0.05, indicating that participants identified more with Player A when they had the same color (*M* = 4.13, SD = 1.42) than a different color (*M* = 3.48, SD = 1.42). A main effect of group membership on the ratings of Player B, *F*(1, 118) = 8.03, *P* = 0.005, partial η^2^ = 0.06, further indicated that participants identified less with Player B in the minimal group condition (*M* = 3.39, SD = 1.03) than in the control condition (*M* = 3.99, SD = 1.31), confirming the effectiveness of our group membership manipulation.

#### Perceived exclusion of target

As expected, the 2 × 2 ANOVA showed a main effect of inclusionary status, *F*(1, 118) = 11.64, *P* = 0.001, partial η^2^ = 0.09. Participants estimated Player B to have received fewer balls in the exclusion game (*M* = 12.78, SD = 5.45) than in the inclusion game (*M* = 15.37, SD = 2.84). The ANOVA also yielded a main effect of group membership, *F*(1, 118) = 6.20, *P* = 0.014, partial η^2^ = 0.05, indicating that participants thought Player B received fewer balls in the minimal group condition (*M* = 13.15, SD = 3.00) than in the control condition (*M* = 15.02, SD = 5.45) irrespective of whether this was the in- or exclusion condition. Because this main effect was unexpected, we conducted follow-up planned comparisons to examine the differences between the specific conditions. These analyses showed that the difference between the minimal group (*M* = 11.59, SD = 2.43) and control conditions (*M* = 13.90, SD = 7.09) was significant in the exclusion condition, *t*(122) = 2.11, *P* = 0.037, Cohen’s *d* = 0.44, 95% CI [.07–2.25], but not significant in the inclusion condition (*M*_minimal group_ = 14.61, SD_minimal group_ = 2.76 *vs M*_control_ = 16.13, SD_control_ = 2.75; *t*(122) = 1.40, *P* = 0.163, Cohen’s *d* = 0.44, 95% CI [−0.31–1.83]). It is not surprising that in the exclusion condition, participants indicated that they thought Player B received fewer balls in the minimal group condition, because this was what actually happened (see results for the percentage of ball tosses to Player B). Although this pattern was not observed in Study 1, these follow-up comparisons thus confirmed that the manipulation of inclusionary status was successful.

#### Ball tosses to target

The 2 × 2 ANOVA showed a significant main effect of inclusionary status, *F*(1, 118) = 11.05, *P* = 0.001, partial η^2^ = 0.09, qualified by a significant interaction, *F*(1, 118) = 4.51, *P* = 0.036, partial η^2^ = 0.04 (see Table 2 for means and s.d.). In the inclusion condition, group membership had no effect on tossing behavior, *t*(122) = −0.65, *P* = 0.519, Cohen’s *d* = 0.17, 95% CI [−0.03–0.01]. In the exclusion condition, however, the percentage of participants’ ball tosses to Player B was lower in the minimal group setting than in the control condition, *t*(122) = 2.34, *P* = 0.021, Cohen’s *d* = 0.57, 95% CI [0.004–0.05]. Mimicking Study 1’s findings, participants actively compensated for Player B’s exclusion in the control condition, with their percentage of tosses exceeding 50% significantly, *t*(30) = 12.20, *P* < 0.001, Cohen’s *d* = 2.50, 95% CI [0.08–0.11], whereas such compensation was only marginally observed in the minimal group condition, *t*(28) = 1.98, *P* = 0.058, Cohen’s *d* = 0.42, 95% CI [−0.002–0.092].

**Table 2:** Percentage of ball tosses to the excluded player as a function of inclusionary status and group membership (Study 2)

	Inclusion		Exclusion	
	*M*	SD	*M*	SD
Minimal group	52.62%^a^	7.90	54.52%^a^	12.29
Control	51.18%^a^	8.75	59.80%^b^	4.47

*Note*: Means with different superscripts differ significantly across all cells (*P*s < 0.05, analyzed with simple-effect analyses).

#### Group identification and tosses to the target

We again correlated difference scores between the perceived group membership of Player A compared to Player B with the ball tosses to Player B. This correlation was significantly negative in the minimal group condition, (*r* = −0.21, *P* = 0.038), but non-significant in the control condition (*r* = −0.15, *P* = 0.223), thereby replicating Study 1.

## Discussion

In Study 2, a minimal group setting was created by automatically assigning participants a color without any accompanying information rather than having participants choose the color themselves, as was done in Study 1. In line with the results from Study 1, this minimal group setting again caused participants to throw fewer balls to an out-group player to the benefit of the in-group player. The correlational results, mimicking those of Study 1, revealed how greater identification with the in-group excluder was again associated with a decrease in throws to the excluded out-group target. Together, this pattern of results across two studies points to the possibility of a motivational conflict that participants might have experienced between including the out-group member on the one hand and favoring the in-group member on the other hand. Still, the reliance on self-report measures and the fact that these assessments were made only after the Cyberball game preclude strong conclusions about the occurrence of such conflict. In a third neuroimaging study, we therefore further investigated whether cognitive conflict arose during participants’ decisions to go along with or compensate for social exclusion in a minimal group setting.

## Study 3

Using a similar setup as in the previous studies, we measured people’s brain activity using fMRI while they played the Cyberball game involving or not the social exclusion of another player when group membership was salient or not. Because our aim was to establish whether motivational conflict would occur when participants had to respond to social exclusion in an intergroup setting, we focused our fMRI analyses specifically on the roles of the dACC and dlPFC, as these regions have reliably been shown to be associated with cognitive conflict (e.g. Van Veen and Carter, 2006).

## Method

### Participants

Our sample was determined at a minimum of *N* = 40, based on recent neuroimaging studies investigating the neural mechanisms underlying social exclusion with a similar experimental setup (Van der Meulen *et al*., 2016, 2017). The final sample consisted of 45 healthy right-handed paid volunteers, who were all students from Leiden University. Due to a technical error during scanning, the data from two participants were lost. We therefore analyzed the data from 43 participants (25 female, 18 male; *M*_age_ = 20.95, SD_age_ = 1.86; age range 18–25). None reported to have any history of neurological or psychiatric disorder and all were medication-free. All participants gave written informed consent for the study, and all procedures were approved by the medical ethical committee of the Leiden University Medical Center (LUMC).

### Design

We used an fMRI-compatible experimental design with one within-subjects factor with two levels (inclusionary status, inclusion *vs* exclusion) and one between-subjects factor with two levels (group membership, minimal group *vs* control). Participants were randomly assigned to the two group membership conditions. We only manipulated group membership in the exclusion game, not in the inclusion game, to avoid habituation to the minimal group manipulation throughout the two subsequent games. In the inclusion game, the three players did not have a color. Our design was therefore not fully factorial. It however still allowed us to compare the effects of group membership (minimal group *vs* control) during the exclusion game and the effects of inclusionary status (inclusion *vs* exclusion) within the minimal group and control conditions separately.

### Procedure

After participants were welcomed and placed in the fMRI scanner, they received instructions about Cyberball. Participants next played two consecutive Cyberball games. In the first game, both other players equally included the other player and the participant. This game was similar to the inclusion game of Studies 1 and 2, but the three players were not colored as to avoid habituation to our minimal group manipulation. As in Studies 1 and 2, we assessed participants’ ball tosses to the target during the game. After the game ended, participants reported the perceived exclusion of the target, by indicating with their left and right index fingers whether they thought the target received more or fewer than 15 ball tosses (i.e. one-third of the total number of ball tosses) from the other player.

Next, participants played the second Cyberball game, which was explained to be with different people from the first game. Now, one player was consistently excluded by the other player (as in the exclusion games of Studies 1 and 2) in either a minimal group setting or control condition, similar to Studies 1 and 2 (see Figure 1A and B). We counterbalanced whether the excluded player was Player A or B, to make sure that inclusion *vs* exclusion behavior was not restricted to the left *vs* right visual field. We again measured participants’ tossing behavior. Following the game, participants again indicated whether they thought the target received more or fewer than 15 ball tosses from the other players. At the end, all participants were asked what they thought the study was about. None of the participants guessed the true purpose of the study or reported any doubts about the cover story.

### fMRI data acquisition

Scanning was performed on a 3.0 T Philips Achieva scanner at the LUMC. Functional data were acquired using a T2*-weighted echo-planar imaging (EPI) sequence (echo time [TE] = 30 ms, repetition time [TR] = 2200 ms, slice matrix = 80 × 80, slice thickness = 2.75 mm, slice gap = 0.28 mm gap, field of view [FOV] = 220 mm), during two fMRI runs which lasted for ~5 min each. At the end of the scan session, a high-resolution T2-weighted anatomical scan (same slice prescription as EPI) was collected.

### fMRI data analysis

Data pre-processing and analysis were conducted with SPM8 software (http://www.fil.ion.ucl.ac.uk/spm/software/spm8) implemented in MATLAB (MathWorks, Sherborn, MA). All functional images were realigned and slice-time corrected using the middle slice as reference. They were spatially normalized to T1 templates and spatially smoothed with a Gaussian kernel (8 mm, full width at half maximum). For motion, we used a cutoff point of 3 mm. None of the participants exceeded this threshold. A canonical hemodynamic response function (HRF) was convolved at the onset of the ball tosses.

Analyses were carried out using the general linear model in SPM8. Whereas the previous fMRI research on targets of exclusion mostly focused on receiving *vs* not receiving the ball, we focused on the brain mechanisms underlying throwing behavior. We focused on throwing behavior, regardless of whether this was to the excluder or the excluded target, because we were interested in brain mechanisms underlying the decision to throw to either one of players. We compared brain activity during these events in the exclusion game (i.e. ExclusionThrow) to the inclusion game (i.e. InclusionThrow), resulting in the ExclusionThrow > InclusionThrow contrast and vice versa. For these contrasts, we subsequently examined the moderating role of group membership by comparing the minimal group condition to the control condition. Although we were less interested in the brain mechanisms involved in the traditionally investigated participant perspective of receiving *vs* not receiving the ball, we nonetheless also compared the brain regions involved in these events separately during the inclusion game (i.e. InclusionGet *vs* InclusionOut) and exclusion game (i.e. ExclusionGet *vs* ExclusionOut), as depicted in Table 3.

**Table 3:** Brain regions revealed by whole-brain contrasts, including MNI coordinates. Peak voxels reported at *P* < 0.001 uncorrected, at least 10 contiguous voxels (voxel size was 3.0 × 3.0 × 3.0 mm)

Anatomical region	L/R	Voxels	*Z*	MNI coordinates			
				*x*	*y*	*z*	
ExclusionThrow > InclusionThrow							
Dorsolateral pre-frontal cortex	L	239	4.66	-27	41	31	^a^
			4.59	-24	53	25	^a^
			4.34	-18	20	34	^a^
InclusionThrow > ExclusionThrow							
Visual cortex	L/R	4874	4.94	39	-85	-8	^b^
			4.92	48	-76	4	^b^
			4.73	45	-46	-23	^b^
[ExclusionThrow > InclusionThrow] Minimal Group >							
[ExclusionThrow > InclusionThrow] Control							
Dorsolateral pre-frontal cortex	L	21	3.70	-24	41	28	
			3.57	-15	50	25	
ExclusionGet – ExclusionOut							
Dorsal anterior cingulate cortex	L/R	580	6.48	-3	-4	52	^b^
			5.37	-6	11	40	^b^
			4.96	-6	17	34	^b^
Motor cortex	L	661	6.95	-42	-22	58	^b^
			6.31	-30	-16	64	^b^
			5.19	-54	-19	46	^b^
ExclusionOut – ExclusionGet							
Visual cortex	L	18	3.89	-39	-16	37	^a^
	R	90	5.74	15	-85	4	^b^
			3.43	6	-82	25	^b^
Motor cortex	L/R	539	6.01	27	-25	64	^a^
			5.61	36	-22	49	^a^
			5.38	-12	-28	67	^a^
InclusionGet > InclusionOut							
Anterior cingulate cortex	L/R	5282	7.12	-6	2	49	^b^
Temporoparietal junction			6.59	-54	-22	34	^b^
Motor cortex			6.91	-33	-10	54	^b^
Insula	L/R	1146	6.70	39	14	7	^b^
			6.56	45	11	7	^b^
			6.51	36	17	10	^b^
InclusionOut > InclusionGet							
Visual cortex	L	53	6.79	-12	-88	1	^b^
	R	187	6.79	15	-85	4	^b^
			6.05	9	-82	22	^b^
			5.80	12	-82	31	^b^

^a^The results remained significant with an FDR-corrected threshold of *P* < 0.05, with an extent threshold of 10 contiguous voxels.

^b^The results remained significant with an FWE-corrected threshold of *P* < 0.05, with an extent threshold of 10 contiguous voxels.

We computed contrast parameter images for each participant and submitted them to second-level group analyses. At the group level, we computed whole-brain contrasts between conditions by performing one-sample *t*-tests, treating participants as a random effect. We further performed two-sample *t*-tests to investigate the moderating role of group membership. Results were considered significant at an uncorrected threshold *P* < 0.001 with an extent threshold of 10 continuous voxels. Thresholds were based on recommendations from Lieberman and Cunningham (2009), to produce a desirable balance between Type I and Type II errors. Table 3 reports which results remained significant with an FDR *P* < 0.05 or FWE *P* < 0.05, >10 contiguous voxel thresholds.

We extracted parameter estimates from the regions that were identified in the whole-brain analyses using the MarsBaR toolbox for SPM8 (Brett *et al*., 2002), to further visualize the patterns of activity.

## Results

### Behavioral results

#### Perceived exclusion of target

The logging of one participant’s estimations failed due to a technical error, leaving 42 participants for this analysis. A Chi-square test of their estimations showed a significant effect of inclusionary status, χ^2^ (1, *N* = 84) = 14.42, *P* < 0.001, φ = 0.41. Participants more often estimated targets to have received fewer than 15 ball tosses in the exclusion game (34 out of 42, 81.0%) than in the inclusion game (17 out of 42, 40.5%). Within the exclusion games, group membership did not further affect these estimations, χ^2^ (1, *N* = 42) = 0.21, *P* = 0.706, φ = 0.07.

#### Participants’ ball tosses to target

Planned comparisons of the number of ball tosses during the two exclusion games showed a significant difference between the minimal group and control condition, *t*(86) = 2.46, *P* = 0.016, Cohen’s *d* = 0.68, 95% CI [0.55–14.67]. Participants’ tosses to the exclusion target were more frequent in the control condition than in the minimal group condition, similar to Studies 1 and 2. In the control condition, participants actively compensated for the target’s exclusion, as their percentage of tosses toward the excluded target significantly exceeded an even 50% distribution, *t*(18) = 7.50, *P* < 0.001, Cohen’s *d* = 1.67, 95% CI [0.11–0.19]. Unlike in Studies 1 and 2, participants in the minimal group condition also tossed the ball to the exclusion target significantly more than 50%, *t*(23) = 2.66, *P* = 0.014, Cohen’s *d* = 0.54, 95% CI [0.02–0.13].

### fMRI results

#### Responses to exclusion

**Fig. 2:**
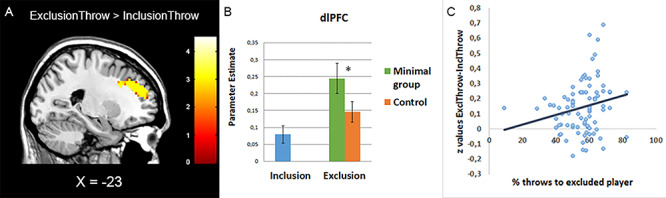
(A) Whole-brain results for regions active in the ExclusionThrow > InclusionThrow contrast (threshold at *P* < 0.05, FDR-corrected). Activation was detected in the dlPFC (MNI coordinates: *x* = −27, *y* = 41, *z* = 31). (B) Parameter estimates plotted for the minimal group and control conditions of the exclusion game and for all participants of the inclusion game (we did not manipulate group membership in the inclusion condition). (C) Activation in the dlPFC correlated positively with the percentage of throws to the excluded player, across all conditions.

**Fig. 3:**
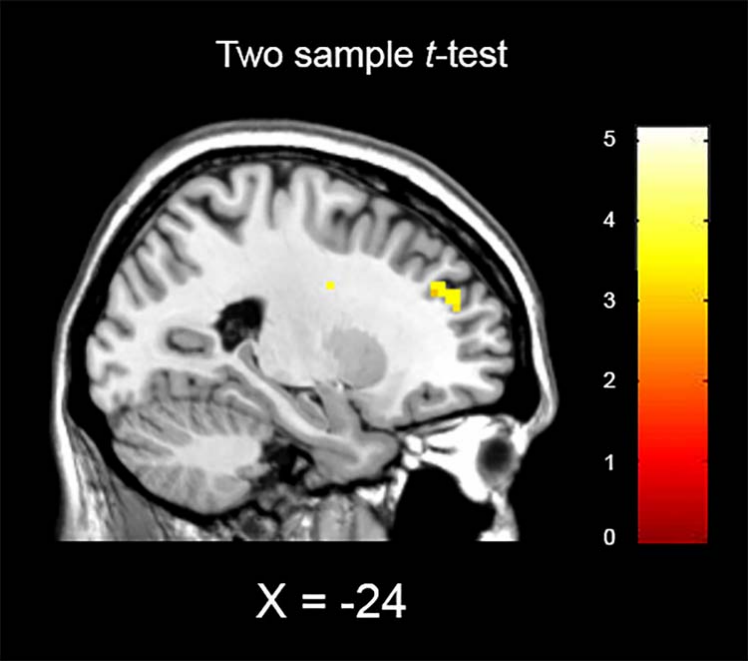
Whole-brain results of the two sample *t*-test for regions active in the ExclusionThrow > InclusionThrow contrast for Minimal Group > Control (threshold at *P* < 0.001, uncorrected). Activation was detected in the dlPFC (MNI coordinates: *x* = −24, *y* = 47, *z* = 28).

The ExclusionThrow > InclusionThrow contrast revealed activation in the dlPFC, which is depicted in Figure 2A and Table 3. We also displayed ROI patterns for this dlPFC activation across different conditions, as depicted in Figure 2B. A paired *t*-test of the parameter estimates revealed that in the exclusion condition (where we manipulated group membership), dlPFC activation was greater for participants in the minimal group condition compared to the control condition, *t*(86) = 2.04, *P* = 0.045, Cohen’s *d* = 0.50, 95% CI [0.002–0.20]. To further examine the effects of group membership, we conducted a two-sample *t*-test comparing ExclusionThrow > InclusionThrow for the Minimal Group > Control contrast. This revealed significantly greater activation in the dlPFC in the minimal group condition compared to the control condition (see Figure 3, Table 3 for all relevant statistics). The reverse contrasts (Control > Minimal group and InclusionThrow > ExclusionThrow) did not reveal any significant activation. None of the whole-brain contrasts revealed increased activation in the dACC.

#### Brain–behavior correlations

To investigate whether the activation in the dlPFC was correlated to participants’ throwing behavior in the Cyberball game, we extracted parameter estimates of our dlPFC region in the ExclusionThrow-InclusionThrow contrast and correlated these with participant’s ball tosses to the target across all conditions. As Figure 2C shows, this correlation was significantly positive, (*r* = 0.21, *P* = 0.048), indicating that the stronger the dlPFC activity, the more frequently participants threw the ball to the excluded target.

## Discussion

Consistent with those of Studies 1 and 2, the findings of Study 3 showed that participants actively included an excluded target in the absence of a minimal group setting. They more often chose not to compensate in a minimal group setting, where an in-group member excluded an out-group target. The fMRI results revealed increased activation in the dlPFC during participants’ tossing behavior in the exclusion game compared to the inclusion game, suggesting that overall, they experienced greater conflict when a fellow player was being excluded. Importantly, however, this activation was stronger in the presence than in the absence of a minimal group setting. This suggests that participants’ throwing decisions while witnessing social exclusion employed greater cognitive control when the exclusion was initiated by an in-group member and the target was an out-group member than when group membership was not made salient. This occurred perhaps to resolve the conflict between two opposing motives, namely, to include others, and to favor the in-group. The results further showed that dlPFC activation correlated positively to inclusion of the target across exclusion conditions. The stronger the dlPFC activity, the more frequently participants threw the ball to the excluded target, suggesting that cognitive control occurred primarily when participants decided to include the target (rather than reciprocate the excluder), further strengthening our conflicting motives account.

Taken together, the results of these three studies provided converging evidence that differences in group membership influence responses to social exclusion of another individual. Still, the use of the modified three-player Cyberball game also created some limitations. In all three studies, the exclusion of the target was directly dependent on the inclusion of the excluder (and vice versa). That is, participants excluded one player by throwing the ball to the other player, who was therefore automatically included in the game. With such a design, it is thus impossible to dissociate people’s exclusion from their inclusion decisions. To address this, we therefore conducted a final study, where we employed a team-selection paradigm without such a direct relation between the inclusion of one person and the exclusion of another person. Moreover, to further examine to what extent our observed pattern of findings could be explained by perceived group membership *vs* reciprocity norms, in Study 4 we directly measured both constructs as potential motivations for participants’ responses to the social exclusion of others. Whereas the reciprocity norm did not seem to motivate participants’ throwing decisions in the absence of a minimal group setting in the previous three studies, these measures would allow us to obtain more direct evidence that participants’ responses to social exclusion within a minimal group setting are primarily motivated by their concerns over group membership. In addition, testing our ideas further with a new task allowed us to extend our findings to a different group context, namely, team selection.

## Study 4

In Study 4, we used a task where participants could adjust the composition of a team by excluding another team member. We adjusted the paradigm from Doolaard *et al.* (2020). In their paradigm participants completed a competitive group task as a group in which the goal was to estimate which of the two dot clouds contain the most dots. Based on the performance of the fellow team members, participants could decide to exclude team members. In our version of the task, we did not give participants feedback on how they performed on the task. Participants first played a practice round, and before the actual game started, participants could choose to adjust the composition of the team by excluding a potential player. We manipulated the group membership of the players in a similar way as in the first three studies, by assigning different colors to the different players to create minimal groups.

In the first three studies, we showed that differences in group membership led participants to throw fewer balls to the excluded target, but only when another player initiated the exclusion. When the other player did not initiate the exclusion, differences in group membership did not lead participants to throw fewer balls to the target. In the current study, we aimed to extend these findings. To do so, we varied the decision order in which participants could choose to exclude another player or not. One-half of the participants were the first in the team to make this decision (the initiate condition). Participants in this condition could thus initiate the exclusion of another player. Based on the findings from the first three studies, we expected that differences in group membership would not lead participants to initiate the exclusion of an out-group player more than an in-group player. The other half of the participants only made the decision to exclude another player after two other team members had already made their decision (the respond condition). In this condition, these other two players always initiated the exclusion of a fourth player, who was to make their choice following the participant. Participants in this condition thus responded to the exclusion of one player that was initiated by two other players. Based on the first three studies, we expected that group membership would influence participants’ exclusion decisions in this condition, such that participants would more often decide to go along with the exclusion initiated by the two other players of an out-group rather than an in-group target. We pre-registered the study’s experimental setup and main hypotheses at https://osf.io/529zf.

### Method

#### Design and participants

The study used a 2 (decision order, initiate *vs* respond) × 2 (group membership, minimal group *vs* control) between-participants design. The previous work using the same paradigm included 40 participants per cell, based on a power analysis that indicated a significant difference with an alpha level of 0.05, a power of β = 0.80 and an effect size of φ = 0.31 (Doolaard *et al*., 2020). Because we incorporated an additional manipulation, we decided to increase the sample size and aimed to collect 50 participants per cell. Due to a logging error, the data from three participants were lost, leaving the data of 197 participants (130 females; *M*_age_ = 37.71, SD = 12.46) for our analyses. Participants were recruited through the online research platform Prolific Academic (https://www.prolific.ac/). To make sure the participants understood our task, we selected only people from the UK and who were native English speakers. All procedures were approved by the ethical committee of the Institute of Psychology of Leiden University.

### Procedure

After giving informed consent, participants were explained that the experiment consisted of a computerized group task, in which participants allegedly formed a team with three other participants. In reality the participants completed the task alone, and the responses of their team members were programmed beforehand. Before starting the actual task, participants learned that each player would be represented by an avatar of a specific color. Depending on the position of their first initial in the alphabet, this would be one out of five colors. After filling out their first initial, participants were presented with their avatar and learned that their avatar was assigned the color orange.

**Fig. 4:**
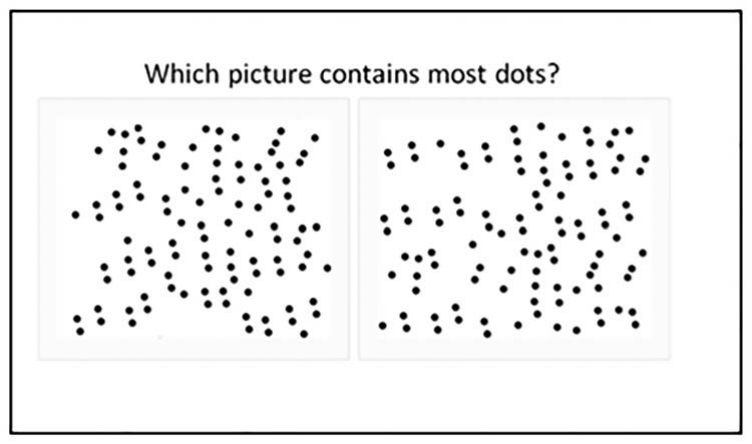
Screenshot of the dot estimation task. Participants selected the picture with most dots.

Participants were then informed that together with their team members, they were going to perform a task in which each participant had to indicate as fast and accurately as possible which of two pictures (see Figure 4) contained the most dots (a procedure similar to the dot-estimation task; see Gerard and Hoyt, 1974). They learned that they played this game against another team and that the team with the highest average team score would win. Participants learned that all team members would first play a practice round of 10 trials. In each trial participants had 10 s to make their decision, after which the next trial was presented. In between trials participants did not receive any feedback about whether they correctly answered the trial or not. Even though participants could not interact with the other members of the group (to avoid that differences in content and valence of the interaction would influence their decisions), we emphasized that like them, the other members of their group also completed the practice round. This was done to create the feeling that participants were really part of a team with which they competed against another group. After the practice round, participants did not receive any feedback on their team members’ or own performance, to make sure performance did not influence their decisions.

**Fig. 5:**
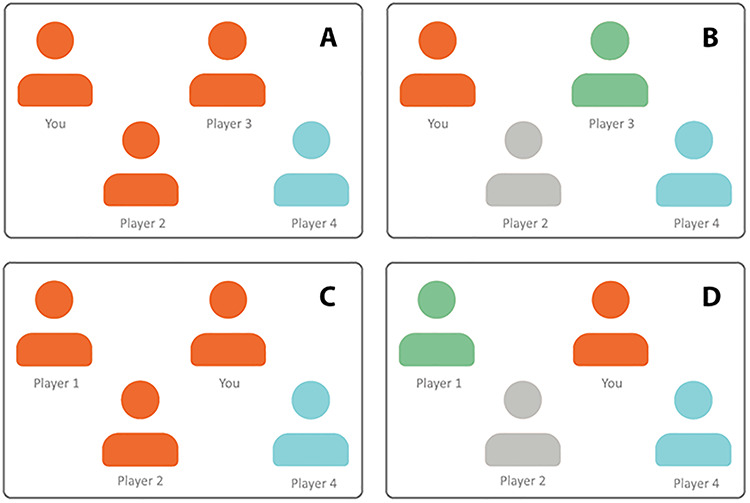
The manipulation of exclusion decision and group membership used in Study 4. Participants were asked whether or not they wanted to adjust the group composition in the presence of a minimal group manipulation (A and C) or in the absence of one (B and D). Moreover, they either had to initiate the decision (initiate condition; A and B) or had to respond to the decision made by other players (respond condition; C and D).

Participants were then told that each team member was asked to indicate with whom they wanted to be in the team in the next round. They were informed that they could choose to be in a team with three or four players. Participants were informed that not the absolute but the average team score achieved in the game would determine whether they would win, and so there was no advantage of choosing to play with four over three team members. Participants then saw a picture with four avatars depicting themselves and their three team members (Figure 5A–D). Similar to the first three studies, in the minimal group condition, two team members were assigned the same color as the participant, and one team member was assigned a different color (see Figure 5A and C). In the control condition, all four team members had a different color (see Figure 5B and D). Depending on the decision order, participants were then told when they could choose with which players they wanted to be in the team. Participants could either initiate the exclusion of one of the other players (initiate condition) or respond to the exclusion of a player initiated by the others (respond condition). In the initiate condition (see Figure 5A and B), participants learned they were Player 1 and were the first to choose their team members, after which Players 2, 3 and 4 would take their turns. In the respond condition (see Figure 5C and D), participants learned they were Player 3 and that they could choose their team members after (seeing the choice of) Players 1 and 2 and before Player 4. Players 1 and 2 would thus make their selection first and always excluded Player 4.

Participants in all conditions were instructed to click once on a player’s icon if they wanted to select that player for the team and twice on the icon of a player if they did not want to have that player in the team for the game. They learned that players who were excluded would receive the message that they had not been chosen to be part of the team and that these players would continue with a different task. This information was added, so that being excluded would not be perceived as an advantage (i.e. finish the experiment early). After making their selection to in-/exclude, participants answered a questionnaire where they had to indicate on a seven-point scale (1 = ‘absolutely not’, 7 = ‘absolutely’) to what extent they agreed with statements about (1) the conflict they experienced, (2) the extent to which reciprocity and group membership motivated their choice and (3) how aversive they were to exclusion.

We measured conflict with two statements (‘I felt torn when deciding on the team composition’ and ‘I experienced conflict when selecting the team players’). Responses were averaged into a single index of conflict (*α* = 0.91). We measured group membership as a motive for participants’ choice with two statements (‘My decision to select a player for the team was based on whether or not the player belonged to my group’ and ‘My decision to exclude a player from the team was based on whether or not the player belonged to my group’). Responses were averaged into a single index of group membership (*α* = 0.81).

Reciprocity was measured only in the respond conditions, because there was no behavior to reciprocate in the initiate condition. We measured reciprocity by asking participants to indicate to what extent they agreed with two statements (‘I did what I did because I was thankful the players before me chose me in their team’ and ‘I based my decision on whether or not the players before me chose me in their team’). Responses were averaged into a single index of reciprocity (*α* = 0.81).

We measured exclusion aversion with two statements (‘I did not like excluding one of the players from the team’ and ‘I found it difficult to exclude one of the players from the team’). Responses were averaged into a single index of exclusion aversion (*α* = 0.79). Finally, to check the manipulation of group membership, we asked participants to what extent they agreed with the statement ‘Player X was a member of my group’, with X being Player 2, 3 or 4 in turn in the initiate condition and Player 1, 2 or 4 in the respond condition. After answering this question about each of the other players, participants were thoroughly debriefed and were paid £0.88.

## Results

### Manipulation check

To establish that our minimal group manipulation had been successful, the manipulation check question to what extent ‘Player X was a member of my group’ had to show differences between the group membership conditions, in particular for Player 4. A 2 × 2 ANOVA on the ratings of Player 4 yielded only a main effect of group membership, *F*(1, 193) = 23.57, *P* < 0.001, partial η^2^ = 0.11, but not of decision order, *F*(1, 193) = 1.41, *P* = 0.237, partial η^2^ = 0.01, and no interaction, *F*(1, 193) = 0.65, *P* = 0.420, partial η^2^ = 0.00. This indicates that irrespective of whether participants were first or followed in their team composition decision, Player 4 was considered to be less of a group member in the minimal group condition (*M* = 3.52, SD = 2.35) than in the control condition (*M* = 5.10, SD = 2.24), thus confirming that our group membership manipulation was successful.

We also examined participants’ responses to the manipulation check for the other two players. Note that in the minimal group condition and the control condition, participants were informed that the other three players—which besides Player 4 also included Players 2 and 3 (in the initiate condition) or Players 1 and 2 (in the respond condition)—were part of their team. It would therefore make sense that participants would overall give high ratings to this question. Indeed, although the means were higher in the minimal group condition (*M* = 6.05, SD = 1.61 and *M* = 6.00, SD = 1.51) than in the control condition (*M* = 5.65, SD = 1.97 and *M* = 5.82, SD = 1.56), 2 (group membership) × 2 (decision order) ANOVAs did not yield any main or interactions effects on these ratings, *F*s < 2.49, *P*s > 0.12.

### Exclusion behavior

To examine the exclusion behavior across conditions, we first conducted a logistic regression analysis with group membership (minimal group *vs* control) and decision order (initiate *vs* respond) as independent variables and participants’ exclusion (yes/no) of the target (Player 4) as the dependent variable. This analysis yielded main effects of group membership, Wald’s χ^2^ (1, *N* = 197) = 13.67, *P* < 0.001, and of decision order, Wald’s χ^2^ (1, *N* = 197) = 13.67, *P* < 0.001. The interaction was not significant, Wald’s χ^2^ (1, *N* = 197) = 0.44, *P* = 0.51. We also analyzed our results with another widely used method to study the interaction effects with a dichotomous dependent variable: the linear probability model (Wooldridge, 2013). A linear probability model is a special case of a binomial regression model, where the probability of observing an event or not (in this case whether participants excluded or not) is treated as depending on one or more explanatory variables. For a detailed analysis of the difference between the linear probability model and binary logistic model, see Hellevik (2009). When analyzing our results with the linear probability model, we do find a significant interaction effect. Results show a significant main effect of decision order (*β* = −0.27, *P* < 0.001) and of group membership (*β* = −0.27, *P* < 0.001), as well as a significant interaction effect (*β* = 0.17, *P* = 0.01).

We then performed follow-up Chi-square tests to investigate the differences between specific conditions. Because the logistic regression interaction effect was not significant, we used a Bonferroni correction and divided *P* = 0.05 by the number of Chi-square tests we performed (i.e. 6). The follow-up Chi-square tests were thus considered significant when *P* < 0.008. In line with our hypotheses, these results showed that participants who responded to the exclusion more often chose to exclude the target in the minimal group condition (22 out of 48, 46.8%) than participants in the control condition (6 out of 50, 12.0%), χ^2^ (1, *N* = 197) = 13.74, *P* < 0.001, φ = –0.37, as well as compared to participants in the minimal group condition who initiated the choice (6 out of 50, 12.0%), χ^2^ (1, *N* = 197) = 13.74, *P* < 0.001, φ = −0.37 and participants in the control condition who initiated the choice (2 out of 49, 4.1%), χ^2^ (1, *N* = 197) = 22.70, *P* < 0.001, φ = −0.48. Chi-square tests between the other three conditions did not yield any significant differences (*P*s > 0.27).

### Decision conflict

A 2 × 2 ANOVA of the conflict ratings yielded a main effect of group membership, *F*(1, 193) = 12.70, *P* < 0.001, partial η^2^ = 0.06, and of decision order, *F*(1, 193) = 63.42, *P* < 0.001, partial η^2^ = 0.25. These main effects were qualified by a significant interaction, *F*(1, 193) = 9.27, *P* = 0.003, partial η^2^ = 0.05. Planned comparisons showed that participants in the minimal group condition who responded to the exclusion experienced more conflict (*M* = 4.68, SD = 1.44) than participants in the control condition who responded to the exclusion (*M* = 3.16, SD = 1.99, *t*(193) = 4.66, *P* < 0.001, *d* = 0.88, 95% CI [−2.16 to −0.88]), who in turn experienced more conflict than participants who initiated the choice in the minimal group condition (*M* = 2.15, SD = 1.37, *t*(193) = 3.14, *P* = 0.002, *d* = 1.80, 95% CI [−1.67 to −0.35]), and in the control condition (*M* = 2.03, SD = 1.57, *t*(193) = 3.49, *P* = 0.001, *d* = 0.63, 95% CI [−1.79 to −0.47]). Participants in the minimal group condition who initiated the choice and participants in the control condition who initiated the choice did not differ significantly in the level of experienced conflict, *t*(193) = 0.37, *P* = 0.713, *d* = 0.08, 95% CI [−0.52 to –0.76]).

### Group membership motive

A 2 × 2 ANOVA of the group membership ratings yielded a main effect of group membership, *F*(1, 193) = 4.43, *P* = 0.037, partial η^2^ = 0.02, and of decision order, *F*(1, 193) = 13.38, *P* < 0.001, partial η^2^ = 0.07. These main effects were qualified by a significant interaction, *F*(1, 193) = 9.62, *P* = 0.002, partial η^2^ = 0.05. Planned comparisons confirmed our predictions that participants who responded to the exclusion indicated that the group membership of the players motivated their team-selection decision more in the minimal group condition (*M* = 3.74, SD = 1.87) than participants in the control condition (*M* = 2.50, SD = 1.51, *t*(193) = 3.67, *P* < 0.001, *d* = 0.73, 95% CI [−1.91 to −0.57]), or participants who initiated the choice in the minimal group condition (*M* = 2.13, SD = 1.61, *t*(193) = 4.77, *P* < 0.001, *d* = 0.92, 95% CI [−2.28 to −0.94]), or the control condition (*M* = 2.37, SD = 1.68, *t*(193) = 4.04, *P* < 0.001, *d* = 0.77, 95% CI [−2.04 to −0.70]). The other conditions again did not differ from one another (*P*s > 0.69).

### Reciprocity motive

Planned comparisons of the reciprocity ratings yielded no significant effect of group membership, *t*(96) = 0.15, *P* = 0.882, *d* = 0.03, 95% CI [−0.53 to 0.46], confirming that the motive to reciprocate the other players did not vary across the minimal group (*M* = 2.57, SD = 1.27) and control conditions (*M* = 2.61, SD = 1.20).

### Exclusion aversion

A 2 × 2 ANOVA of participants’ exclusion aversion ratings yielded no main effects of group membership, *F*(1, 193) = 0.06, *P* = 0.814, partial η^2^ = 0.00, or decision order, *F*(1, 193) = 1.29, *P* = 0.257, partial η^2^ = 0.01, and no interaction, *F*(1, 193) = 0.36, *P* = 0.550, partial η^2^ = 0.00. Across conditions participants indicated to be relatively exclusion averse, with an overall mean score that was above the midpoint of the seven-point scale (*M* = 4.69, SD = 1.94).

### Mediated moderation

We explored whether the interaction effect of group membership and decision order on participants’ exclusion behavior would be mediated by experienced conflict and/or by group membership motives. To examine this, we performed a mediated moderation analysis (Muller *et al*., 2005). To test this, we used Hayes’s (2018) PROCESS bootstrapping command with 10 000 iterations (model 8) to test the indirect effect (Preacher *et al*., 2007) of the interaction term of group membership and decision order on exclusion behavior through experienced conflict and/or group membership motives (controlling for the unique effects of group membership and decision order). The analysis revealed a significant indirect effect of group membership motives on exclusion behavior (the 95% CI did not contain zero, *a* × *b* = 0.66, SE = 0.32, 95% CI [0.20–1.46]), but not of experienced conflict (the 95% CI did contain zero, *a* × *b* = −.13, SE = 0.27, 95% CI [−0.73–0.37]). The findings thus suggest that group membership motives can explain the effects of group membership on participants’ decisions in response to the exclusion by the other players. However, because of the exploratory nature of this analysis, these findings should be interpreted with caution.

## Discussion

Employing a different paradigm, within a different group setting, the findings of Study 4 further supported the results of our first three studies that a simple minimal group manipulation made participants compensate less for the exclusion of an out-group target. When deciding on whom to select for a team, participants more often decided to go along with the exclusion by another player in the presence than in the absence of a minimal group. When participants initiated the decision, they excluded less often, regardless of the presence or absence of a minimal group. We investigated several motives for participant’s choices. Self-report ratings of experienced conflict showed that responding to the exclusion of an out-group member that was initiated by an in-group member increased the experience of conflict, converging with the fMRI results of Study 3. In line with these findings, the results showed that even when participants decided to go along with the exclusion of an out-group target, they still indicated (across all conditions) to be exclusion averse. Moreover, when participants responded to the exclusion of an out-group player, they also indicated that their decision to exclude or not was based on whether or not the player belonged to their group. Finally, reciprocity motives did not play a role in participants’ responses to social exclusion. Mediated moderation analyses showed that although experienced conflict was higher when participants excluded more, conflict did not predict exclusion behavior significantly. Therefore, though conflict occurs, it may not be the essential mechanism that drives the exclusion behavior. Instead, the analysis showed that participants’ exclusion decisions were motivated by the group membership of the players.

## General discussion

In three behavioral studies and one behavioral fMRI study using different experimental paradigms, we investigated the effect of group membership on participants’ responses to the social exclusion of others, by varying the absence or presence of a minimal group setting. In the first three studies, we employed a modified version of the three-player Cyberball game and examined participants’ ball tosses to an excluded target in the absence or presence of a minimal group setting. In these studies participants actively included an excluded target in the absence of a minimal group setting (i.e. increased the number of tosses toward), but chose not to intervene when an in-group member excluded an out-group target (i.e. distributing their tosses more or less evenly). Although participants did not fully exclude the out-group target in this case, the result of their indecisive behavior was that, compared to the other players, the target received significantly fewer balls. Correlation results from Studies 1 and 2 moreover showed that the more participants identified with the excluder than the excluded target in a minimal group setting, the less frequently they compensated by again throwing the ball to the excluded target, which suggests that participants experienced a motivational conflict between favoring the in-group and avoiding the exclusion of the out-group target.

Note though that in the three-player Cyberball setting of our first three studies, throwing the ball to the excluder automatically ruled out a throw to the excluded player, making it difficult to disentangle inclusion of the in-group member from exclusion of the out-group member. In a fourth study, we therefore employed a different paradigm that allowed us to dissociate these responses. In this paradigm, participants could adjust the composition of a team by in- *vs* excluding players from an initial group of four, while we again manipulated group membership through the absence or presence of a minimal group setting. The results of this study showed that whereas participants were exclusion averse in the absence of a minimal group setting, they decided to actively exclude out-group targets when this was initiated by an in-group member. Mediated moderation analyses showed that group membership motives accounted for this decision to go along with the exclusion. In addition, Study 4 allowed us to replicate the Cyberball findings of the first three studies in a different group setting, namely, team selection.

In addition to self-reports and behavioral measures, our third study also employed neuroimaging that allowed us to assess through more implicit measures to what extent participants experienced conflicting motives while deciding to exclude or not. These neuroimaging findings revealed that during the exclusion game compared to the inclusion game, activation increased in the dlPFC, a brain region widely associated with the resolution of cognitive conflict (Van Veen and Carter, 2006). Importantly, this relative activation was even stronger in the presence than in the absence of a minimal group setting, suggesting that participants’ throwing decisions following exclusion concurred with greater cognitive control in response to conflict when the exclusion was initiated by an in-group member than in the absence of a minimal group setting. Further analyses moreover revealed that dlPFC activation was positively correlated to compensation. The stronger the dlPFC activity, the more frequently the participants threw the ball to the excluded target, suggesting that cognitive conflict was primarily present when participants decided to override the tendency to reciprocate the excluder and instead again include the exclusion target. Finally, the self-report measures in Study 4 supported the fMRI findings, showing that participants experienced more conflict when they responded to the exclusion of a target in the presence than in the absence of a minimal group manipulation. Moreover, when participants decided to go along with the exclusion of an out-group target, they still indicated to be exclusion averse, suggesting that different motives have affected participant’s decisions.

Although together these findings suggest that participants experienced conflict when they responded to the exclusion of an out-group target initiated by an in-group member, the mediation analysis in Study 4 showed that this self-reported conflict was not associated with their exclusion decisions. Note though that participants experienced self-reported conflict only after the team-selection task had already been completed. Perhaps then, through their decision, they had already resolved this conflict, irrespective of whether this involved going along with the exclusion of the out-group target or not. Experienced conflict may therefore not have affected participants’ exclusion decisions. Instead, our mediation analysis showed that whether or not participants went along with the exclusion of an out-group target could be explained by group membership motives. That is, participants’ decision to exclude a player or not was based on whether or not the player belonged to their group.

Our neuroimaging findings from Study 3 showed no association of participants’ responses to social exclusion with the ACC, even though this was one of our regions of interest. The previous work on the involvement of the ACC and dlPFC in conflict adaptation suggests that the ACC is primarily associated with conflict monitoring whereas the dlPFC is more involved with conflict resolution (Kerns *et al*., 2004; Kerns, 2006; Smith *et al*., 2019). More recent work using lesion patients showed that while the dlPFC plays a fundamental role in behavioral adaptation in response to conflict, the ACC is sensitive to the level of conflict, but is not crucial for handling conflict (Boschin *et al*., 2017). Along those lines, the dlPFC may have guided participants’ ball tosses more than the ACC. At this point, the above interpretation is still speculative in nature, and future research is required to further establish the role of the dlPFC in participant’s responses to exclusion in our studies.

### Limitations and implications

A limitation of our first three studies was that participants were faced with a dilemma where the inclusion of one player was directly linked to the exclusion of the other player. That is, when participants decided to throw the ball to the out-group target, they at the same time excluded their in-group member, and vice versa. In daily life, many decisions involve such dilemmas (e.g. whom to work with on an assignment, whom to pass the ball to in a game of soccer, whom to talk to at a party). We were therefore specifically interested in how people deal with this tension between the inclusion of one person and at the same time the exclusion of another person and how group membership affects this decision-making process. However, to disentangle these decisions, we also replicated our findings with a different paradigm, where participants responded to social exclusion in a situation without a direct relation between the exclusion of one player and the inclusion of another.

Another limitation is the focus on social exclusion dynamics in relatively small groups. In the current studies, we measured people’s responses to the social exclusion of others in a three-person (Studies 1–3) or a four-person (Study 4) interaction. We, however, have no reason to expect that our findings are restricted to these smaller groups. Previous research has shown that even in larger groups, participants still notice and respond to the exclusion of a fellow group member (Jones *et al*., 2019). Future research could investigate to what extent group membership also plays a moderating role in the responses to social exclusion in larger groups.

Our mediation analyses in Study 4 showed that participants’ decision to exclude a player or not was based on whether or not the player belonged to their group. However, in the absence of a direct comparison of the participants’ attitudes toward in-group *vs* out-group players, it is difficult to conclude whether in-group favoritism or out-group derogation explains participant’s exclusion behavior in our studies. It is, however, important to note that whereas group membership affected participants’ reactions to social exclusion, it did not affect participants’ ball tosses in the inclusion game (in Studies 1 and 2). If participants by default had the intention to exclude an out-group member, our minimal group manipulation would have resulted in participants throwing the ball more to the in-group player, independent of inclusionary status (i.e. in both the inclusion and exclusion conditions). Moreover, in Study 4 we showed that when participants were the first to decide, they rarely decided to exclude the out-group target. Only when the other players initiated the exclusion, participants decided to exclude the target. Together, these findings thus more likely reflect in-group favoritism (including the in-group member; Greenwald and Pettigrew, 2014) rather than out-group derogation (excluding the out-group target; Yamagishi and Kiyonari, 2000; Halevy *et al*., 2008).

Participants in our four studies compensated less for the exclusion of a target when the target was an out-group member than when group membership was not made salient. Although these findings fit with literature demonstrating that people are less cooperative with out-group members than with in-group members (Goette *et al*., 2006; Balliet *et al*., 2014), and share fewer resources with out-group members (Chen and Li, 2009; Baldassarri and Grossman, 2013), they diverge from a recent four-player Cyberball study involving adolescents (Vrijhof *et al*., 2016). In this study it was found that, regardless of whether the excluded target was an in- or out-group member, adolescents actively included the excluded target. One explanation for the differences between their findings and our own results could be the difference in age groups. Perhaps our adult participants were more affected by the minimal group manipulation than the adolescent participants. Another explanation could be that variations in design and procedure explain the differences with Vrijhof *et al.* (2016). Because all participants in their study also played four other versions of the Cyberball game, where group membership was manipulated differently or not at all, participants’ behavior in the fifth game (which resembled our current studies) may have been influenced by the norm that had already emerged during the four previous games. Future research could examine whether our findings among adults would hold up in a design that more closely resembles Vrijhof *et al.* (2016) or, reversely, in adolescents when the design more closely resembles that of our Studies 1–3. Indeed, their findings did reveal that adolescents were showing greater empathic concern for in-group compared to out-group players, suggesting that the minimal group manipulation did affect their affective responses.

Somewhat unexpected, and unlike in Cyberball Studies 1 and 2, the participants in Cyberball Study 3 tossed the ball to the exclusion target significantly more than 50% in the minimal group condition, thus compensating the exclusion by the other player. Because our neuroimaging-compatible setup required that all participants first played an inclusion game (without a group membership manipulation) before they proceeded with the exclusion game, they may have been more inclined to transfer this inclusion norm to the secondary exclusion game (much like the adolescents in the study by Vrijhof *et al*., 2016). The within-subjects design of Study 3 may thus have weakened the effect of our minimal group manipulation. Future research could investigate whether the differences between the third and the previous two Cyberball studies can be explained by the transfer of an inclusion norm across games.

## Conclusion

Most research on social exclusion has focused strongly on its detrimental effects on victims (Williams, 2007). In the last decade, however, research has begun to also examine the sources of exclusion (for a special issue, see, e.g. Volume 155, Issue 5 of the *Journal of Social Psychology*) and, more specifically, the actors involved. As a result, new paradigms have been developed to investigate the actors of social exclusion. The current studies add to this emerging research perspective by focusing on individuals who respond to social exclusion that is initiated by other group members (see also Riem *et al*., 2013; Van der Meulen *et al*., 2016, 2017; Vrijhof *et al*., 2016) and further underline the importance of taking the group dynamics into account when examining the emergence of social exclusion (Nezlek *et al*., 2012; Poulsen and Carmon, 2015).

The current research is the first to show that when in-group members initiate the exclusion of an out-group member, either people choose not to intervene and consequently fail to adequately compensate for this exclusion (Studies 1–3) or people choose to go along with the exclusion as to favor their in-group but experience increased levels of conflict (Study 4). Irrespective of whether people act in a more passive manner or choose to actively jump on the ‘bad’wagon, the consequence of their behavior is relative exclusion of the target. Our findings thus stress the importance of involving all members of a group when studying social exclusion behavior, according with social–ecological theories highlighting the role of peers, colleagues, teachers, and families (Swearer and Espelage, 2004; Williams, 2007). Viewing social exclusion as a group dynamic, rather than a social interaction between an actor and victim dyad, allows educators and researchers to think about prevention and intervention efforts that include all individuals within a group, as minimal as this group may be.

## Conflict of interest statement

The authors declare that there is no conflict of interest.

## Supplementary Material

scan-18-419-File010_nsaa070

## Notes

^1^In all four studies there were no differences in relevant demographics between the different groups. Gender and age were equally distributed across conditions.^2^We also examined whether participants chose to exclude the other two players who were not the target (Players 2 and 3 in the initiate condition and Players 1 and 2 in the respond condition). In none of the conditions, more than 3 participants chose to exclude one of these players. There were no significant differences between conditions.
