# Supplementary material for: Jumping on the ‘bad’wagon? How group membership influences responses to the social exclusion of others
Source: Soc Cogn Affect Neurosci. 2020 May 21;15(5):571–86. doi: 10.1093/scan/nsaa070 (PMC7328018; doi:10.1093/scan/nsaa070)
Supplement: scan-18-419-File010_nsaa070 [file scan-18-419-file010_nsaa070.docx]

**Supplemental material**

Table S1: *Additional measures not reported in the manuscript, including results, as a function of inclusionary status (IS) and group membership (GM), Study 1*

| *Questions* |  | *Means (SDs) and ANOVA results* | | | | | |
| --- | --- | --- | --- | --- | --- | --- | --- |
|  | Inclusion | | Exclusion | |  | Statistics | |
|  | Minimal group | Control | Minimal Group | Control |  | *F*(1,122) | *p* |
| 1. During the game each player was equally included | 4.39(2.32) | 4.07(1.86) | 4.47(2.30) | 3.94(2.19) | IS  GM  IS × GM | .00  1.20  .07 | .96  .28  .79 |
| 1. During the game Player A was included in the group | 6.00(1.13) | 5.06(1.50) | 5.83(1.23) | 5.26(1.54) | IS  GM  IS × GM | .01  9.49  .56 | .95  .00  .45 |
| 1. During the game Player B was included in the group | 6.06(.93) | 5.29(1.24) | 4.57(1.87) | 4.29(1.82) | IS  GM  IS × GM | 21.10  3.72  .85 | .15  .03  .01 |
| 1. During the game Player A felt bad | 2.13(1.36) | 2.29(1.10) | 2.47(1.28) | 2.21(1.25) | IS  GM  IS × GM | .32  .05  .90 | .57  .82  .35 |
| 1. During the game Player B felt bad | 2.55(1.67) | 2.48(1.34) | 4.07(1.89) | 3.26(1.91) | IS  GM  IS × GM | 14.00  1.99  1.44 | .00  .16  .23 |
| 1. During the game I felt included in the group | 4.39(1.82) | 4.42(1.50) | 5.73(1.55) | 6.00(1.39) | IS  GM  IS × GM | 27.31  .29  .18 | .00  .60  .68 |
| 1. During the game I felt good | 4.52(1.75) | 4.71(1.68) | 5.40(1.61) | 5.24(1.60) | IS  GM  IS × GM | 5.68  .00  .37 | .02  .96  .55 |
| 1. During the game I felt bad | 3.10(1.80) | 2.87(1.54) | 2.73(1.60) | 2.26(1.54) | IS  GM  IS × GM | 2.81  1.44  .18 | .10  .23  .68 |
| 1. During the game Player A was unkind to the other players | 2.90(1.83) | 2.81(1.47) | 4.70(1.80) | 3.91(2.22) | IS  GM  IS × GM | 19.11  1.78  1.09 | .00  .19  .30 |
| 1. During the game Player B was unkind to the other players | 3.13(1.89) | 2.87(1.54) | 2.63(1.35) | 2.65(1.59) | IS  GM  IS × GM | 1.57  .18  .22 | .21  .67  .64 |

Table S2: *Additional measures not reported in the manuscript, including results, as a function of inclusionary status (IS) and group membership (GM), Study 2*

| *Questions* |  | *Means (SDs) and ANOVA results* | | | | | |
| --- | --- | --- | --- | --- | --- | --- | --- |
|  | Inclusion | | Exclusion | |  | Statistics | |
|  | Minimal group | Control | Minimal Group | Control |  | *F*(1,122) | *p* |
| 1. During the game each player was equally included | 4.45(1.91) | 4.45(2.14) | 3.72(2.12) | 4.45(2.26) | IS  GM  IS × GM | .90  .90  .90 | .35  .35  .35 |
| 1. During the game Player A was included in the group | 5.26(1.59) | 5.48(1.34) | 5.17(1.07) | 5.03(1.82) | IS  GM  IS × GM | 1.00  .03  .46 | .32  .87  .50 |
| 1. During the game Player B was included in the group | 4.97(1.60) | 5.68(1.08) | 4.24(1.43) | 4.58(1.98) | IS  GM  IS × GM | 10.43  3.45  .43 | .00  .07  .51 |
| 1. During the game Player A felt bad | 2.58(1.23) | 2.45(1.18) | 2.55(1.33) | 2.77(1.23) | IS  GM  IS × GM | .43  .04  .61 | .52  .84  .44 |
| 1. During the game Player B felt bad | 2.87(1.57) | 2.52(1.34) | 3.72(1.69) | 3.29(1.40) | IS  GM  IS × GM | 8.97  2.11  .02 | .00  .15  .89 |
| 1. During the game I felt included in the group | 4.68(1.54) | 4.58(1.79) | 5.76(1.15) | 5.90(1.25) | IS  GM  IS × GM | 20.75  .01  .21 | .00  .93  .65 |
| 1. During the game I felt good | 4.65(1.38) | 4.55(1.65) | 5.03(1.57) | 5.29(1.27) | IS  GM  IS × GM | 4.49  .09  .44 | .04  .77  .51 |
| 1. During the game I felt bad | 2.94(1.39) | 3.00(1.57) | 2.62(1.42) | 2.16(1.04) | IS  GM  IS × GM | 5.42  .64  1.12 | .02  .43  .29 |
| 1. During the game Player A was unkind to the other players | 2.68(1.68) | 2.90(1.51) | 4.10(2.24) | 3.94(2.16) | IS  GM  IS × GM | 12.52  .01  .32 | .00  .93  .57 |
| 1. During the game Player B was unkind to the other players | 2.71(1.51) | 2.84(1.59) | 3.03(1.45) | 3.10(1.87) | IS  GM  IS × GM | .99  .11  .01 | .32  .74  .91 |

Table S3: *Additional measures not reported in the manuscript, including results, as a function of inclusionary status (IS) and (for the exclusion conditions only) group membership (GM), Study 3*

| *Questions* |  | *Means (SDs) and planned comparison results* | | |
| --- | --- | --- | --- | --- |
|  | Inclusion | | Exclusion | |
|  |  | | Minimal Group | Control |
| 1. During the game I felt like I belonged | 3.00(1.35)^a^ | | 4.06(1.11)^b^ | 4.63(.49)^b^ |
| 1. I felt accepted by the other players | 3.30(1.17)^a^ | | 4.28(.83)^b^ | 4.71(.46)^b^ |
| 1. I thought the other players determined everything | 2.58(1.20)^a^ | | 1.61(.50)^b^ | 1.67(.70)^b^ |
| 1. I felt like I had control over the game | 2.74(1.11)^a^ | | 3.83(1.15)^b^ | 4.21(.83)^b^ |
| 1. I had a good feeling about myself during the game | 3.53(1.03)^a^ | | 3.89(.76)^ab^ | 4.17(.70)^b^ |
| 1. My self-esteem was high during the game | 3.19(.91)^a^ | | 3.83(.71)^b^ | 4.08(.83)^b^ |
| 1. I felt invisible during the game | 2.02(1.12)^a^ | | 1.44(.78)^ab^ | 1.29(.55)^b^ |
| 1. I felt like my existence had a purpose during the game | 3.12(1.12)^a^ | | 3.33(1.14)^a^ | 3.71(1.08)^a^ |
| 1. I felt good during the game | 3.33(1.13)^a^ | | 3.67(.84)^b^ | 4.29(.81)^c^ |
| 1. I felt bad during the game | 2.02(1.08)^a^ | | 1.50(.71)^a^ | 1.67(.92)^a^ |
| 1. How do you think Player A experienced the game (1 = *negative*; 5 = *positive*) | 4.05(.69)^a^ | | 3.61(1.04)^a^ | 3.58(.93)^a^ |
| 1. How do you think Player B experienced the game (1 = *negative*; 5 = *positive*) | 3.95(.75)^a^ | | 3.33(1.08)^ab^ | 3.00(1.41)^b^ |
| 1. To what extent was Player A’s behavior justified? | 3.21(.86)^a^ | | 2.94(1.00)^a^ | 3.04(1.27)^a^ |
| 1. To what extent was Player B’s behavior justified? | 3.19(.82)^a^ | | 3.39(.85)^a^ | 3.54(.83)^a^ |
| 1. To what extent was Player A’s behavior fair? | 3.05(1.02)^a^ | | 3.00(1.14)^a^ | 3.08(1.44)^a^ |
| 1. To what extent was Player B’s behavior fair? | 3.21(1.01)^a^ | | 3.56(.92)^a^ | 3.79(1.06)^a^ |

*Note*. Means with different superscripts differ significantly within rows (*p*s <.05, analyzed with planned contrasts).
